# Guided blood transfusion of trauma patients with rotational thromboelastometry: a single-center cohort study

**DOI:** 10.1186/s13017-023-00508-5

**Published:** 2023-07-01

**Authors:** Mina Salehi, Rajan Bola, Nenke de Jong, Andrew W. Shih, Naisan Garraway, Philip Dawe

**Affiliations:** 1 Division of General Surgery, Trauma and Acute Care Surgery, Vancouver General Hospital, University of British Columbia, Jim Pattison Pavilion, 855 W 12th Ave, Vancouver, BC V5Z 1M9 Canada; 2 Canadian Armed Forces, 1 Canadian Field Hospital, 147 Flanders Row, Garrison Petawawa, ON K8H 2X3 Canada; 3 Department of Pathology and Laboratory Medicine, Division of Hematopathology, Vancouver General Hospital, University of British Columbia, Jim Pattison Pavilion, 899 W 12th Ave, Vancouver, BC V5Z 1M9 Canada

**Keywords:** Blood products, ROTEM, Transfusion, Trauma

## Abstract

**Background:**

Rotational thromboelastometry (ROTEM) is a blood test used to measure in vitro clot strength as a surrogate for a patient’s ability to form clots in vivo. This provides information about induction, formation, and clot lysis, allowing goal-directed transfusion therapy for specific hemostatic needs. We sought to evaluate the effect of ROTEM-guided transfusion on blood product usage and in-hospital mortality among patients with a traumatic injury.

**Methods:**

This was a single-center observational cohort analysis of emergency department patients in a Level 1 trauma center. We compared blood usage in trauma patients in whom ratio-based massive hemorrhage protocols were activated in the twelve months before the introduction of ROTEM (pre-ROTEM group) to the twelve months following the introduction of ROTEM (ROTEM-period group). ROTEM was implemented in this center in November 2016. The ROTEM device allowed clinicians to make real-time decisions about blood product therapy in resuscitation for trauma.

**Results:**

The pre-ROTEM group contained 21 patients. Forty-three patients were included from the ROTEM-period, of whom 35 patients received ROTEM-guided resuscitation (81% compliance). The use of fibrinogen concentrate was significantly higher in the ROTEM-period group (pre-ROTEM mean 0.2 vs. ROTEM-period mean 0.8; *p* = 0.006). There was no significant difference in the number of units of red blood cells, platelets, cryoprecipitate, or fresh frozen plasma transfused between these groups. There was no significant difference in the mortality rate between the pre-ROTEM and ROTEM-period groups (33% vs. 19%; *p* = 0.22).

**Conclusions:**

The introduction of ROTEM-guided transfusion at this institution was associated with increased fibrinogen usage, but this did not impact mortality rates. There was no difference in the administration of red blood cell, fresh frozen plasma, platelet, and cryoprecipitate. Future research should focus on increased ROTEM compliance and optimizing ROTEM-guided transfusion to prevent blood product overuse among trauma patients.

**Supplementary Information:**

The online version contains supplementary material available at 10.1186/s13017-023-00508-5.

## Background

Trauma remains one of the leading causes of death in adults [1, 2]. Nearly all deaths after trauma occur within the first 24 h, and uncontrolled bleeding is the most significant cause of preventable death in severe traumatic injuries [3, 4]. Up to 35% of trauma patients develop coagulopathy, despite advances in trauma systems, resuscitation, and management of these patients. This is associated with a mortality rate of up to 40% [5–8]. Acidosis, hypothermia, and hemodilution, also known as the triad of death, may exacerbate trauma-induced coagulopathy due to resuscitation efforts.

Damage control resuscitation has improved the outcome of severe bleeding trauma patients [9–11]. This practice is based on empirically transfusing trauma patients with red blood cells (RBC), fresh frozen plasma (FFP) and platelets in a 1:1:1 ratio, originating from early studies in military trauma mimicking whole blood transfusion [10]. Likewise, directed fibrinogen replacement therapy, which is primarily indicated for congenital and acquired fibrinogen deficiency, such as liver failure or disseminated intravascular coagulopathy, has been shown to produce favorable survival rates in traumatized patients, but is not commonly used in current transfusion practices in North America [12, 13].

The standard coagulation laboratory tests to assess the hemostatic function of the patient include the international normalized ratio (INR), prothrombin time (PT), activated partial thromboplastin time (APTT), and fibrinogen and platelet count. However, many conventional coagulation tests were not designed to assess coagulation status in the bleeding patient (INR, PT, and APTT). These tests may also be associated with delays in therapy due to variability in testing turnaround times among centers. Point-of-care coagulation testing, such as viscoelastic assays of hemostasis, has been shown to better predict coagulation status [14], largely due to determining many functional aspects of the clot [15]. Thromboelastography and rotational thromboelastometry (ROTEM) are two examples of viscoelastic assays that have been used to assess the hemostatic status in a broad spectrum of bleeding patients. Several studies have determined a significant and clinically relevant correlation between ROTEM parameters and coagulopathy in trauma patients [16, 17].

While balanced resuscitation has been adopted for initial, empiric resuscitation, ROTEM can identify clot formation, strength, or degradation problems that can guide targeted therapy to improve hemostasis. This may lower overall product usage and thereby decrease transfusion-related adverse events and improve trauma-related mortality. Use of ROTEM to guide transfusions to correct coagulopathy has been associated with decreased post-operative bleeding in other settings such as cardiac surgery and may also reduce blood product usage [18].

The purpose of this study was to evaluate the effect of the implementation of ROTEM-guided transfusion on blood product transfusion and mortality. We propose that blood product transfusions in ROTEM-period resuscitation will differ from pre-ROTEM resuscitation. Our secondary hypothesis was that the use of ROTEM would reduce the mortality in traumatic injury.

## Methods

### Study design and setting

This was a single-center study in a Level 1 trauma center to determine the effect of the implementation of ROTEM on our resuscitation practices by comparing one year before the implementation of ROTEM to one year after the implementation. We used an observational cohort design. All patients who are 18 years of age or older in whom our center’s ratio-based massive hemorrhage protocol, known as the Trauma Exsanguination Protocol (TEP), was activated twelve months prior to the implementation of ROTEM were included in the pre-ROTEM-group. The ROTEM device has been available for use in trauma patients since November 2016. We prospectively included all trauma patients in whom the TEP was activated in the following twelve months. Patients who did not sustain traumatic injuries were excluded.

### Trauma exsanguination protocol

Transfusion in trauma patients at our institution was guided by the TEP and laboratory values, including conventional coagulation testing and ROTEM after its introduction in our center. The TEP is activated by the Trauma Physician based on clinical discretion. Upon initiation of TEP, the blood bank will immediately supply a ‘four’-pack (four units RBC and four units FFP). This is followed with ‘six’-packs (six units RBC, six units FFP and one dose of platelets equivalent to four units of platelets) empirically until laboratory testing results are available. Patients’ resuscitation is then tailored using the result of ongoing laboratory tests of hemoglobin, platelet count, INR, fibrinogen, and calcium. The aim is to achieve a 1:1:1 ratio for RBC:FFP:Platelets after the initial four units of RBC. In addition, patients were administered tranexamic acid as part of the protocol, given as a 1-g bolus followed by another 1-g bolus if there were signs of ongoing bleeding or if indicated by ROTEM. The TEP is ended at the discretion of the trauma physician.

### Rotational thromboelastometry

ROTEM is a whole blood coagulation test that measures in vitro clot strength as a surrogate for a patient’s ability to form a clot; ordered with all TEPs as part of standard of care. The ROTEM device offers online results to the trauma physician within 15 min of TEP activation. At our institution, the ROTEM device is housed in the laboratory rather than the clinical areas to assist in standardization and to allow multiple clinical services to access ROTEM testing. However, results are available in real-time online from any computer in the hospital to allow clinicians to make time-sensitive decisions about blood product therapy in resuscitation for trauma. The TEP is followed with ROTEM results allowing for targeted therapy of specific deficiencies.

The standard normal values for ROTEM parameters used in the study institution are Extem amplitude at 10 min (A10) > 50 mm, Extem clotting time (CT) < 95 s, Extem maximum lysis (ML) 30 < 10% and Fibtem A10 > 10 mm. Transfusions are guided by an institutional algorithm (Additional file 1: Fig. S1). If the Extem A10 was 40 mm or less (and/or the clot formation time was greater than 130 s), with a Fibtem A10 10 mm or greater, an adult dose of platelets was recommended; if the Fibtem A10 was less than 10 mm, fibrinogen replacement was recommended. Elevations of the Extem CT are recommended to have frozen plasma transfusion to correct coagulation factor deficiencies. The study institution also performs a Clauss fibrinogen assay, where low fibrinogen is considered a fibrinogen level under 1.5 mg/L. Coagulopathy of trauma is considered as an INR above 1.2 [19].

### Data collection

Data were collected from chart review to include patient demographics, injury characteristics, laboratory results, transfusion data, and in-hospital mortality. We retrospectively collected data on the group before the implementation of ROTEM (pre-ROTEM group) and prospectively collected data for the patients after implementation of ROTEM (ROTEM-period group). The blood samples for ROTEM were collected upon arrival of the patient in the emergency department and analyzed by the hematology laboratory. The relevant parameters assessed by the clinicians included CT, CFT, A10, maximum clot firmness (MCF), alpha angle (alpha) and ML 30 to allow for goal-directed transfusion therapy in the trauma patient. The primary outcome of interest was the number of allogeneic blood components and blood products used. The secondary outcome of interest was in-hospital mortality.

### Data analysis

Values are reported as mean with standard deviation (SD) or median with interquartile range [IQR] if distributions are skewed, raw percentages, and frequencies with associated percentages for categorical variables. The cohort was analyzed using the unpaired Student t test for continuous variables or the Mann–Whitney U test for nonparametric data, and categorical variables were analyzed using Pearson’s *χ*^2^ test. Data with a *p* value < 0.05 were considered significant. The groups were analyzed for all variables collected. The data were analyzed with the use of SPSS statistical software (Version 25, Chicago, USA).

## Results

During the two-year study period, a total of 64 patients were included in the analysis (Table 1). Of these, 21 patients (33%) had a TEP activated in the pre-ROTEM-period and 43 patients (67%) in the ROTEM-period. Patient demographics such as age, sex, mechanism of injury, and injury severity score (ISS) were similar between the two groups. The median age of the population was 42 [30–55], and most patients (84%) were male. The majority of the population sustained blunt trauma (79%). The median ISS was 33 [18–43]. 27% of patients were admitted to the hospital with signs of trauma-induced coagulopathy (INR > 1.2).

**Table 1:** Patient demographics comparing pre-ROTEM versus ROTEM-period

Variable	Pre-ROTEM n = 21	ROTEM-period *n* = 43	*p* value
Age, years	41 [25]	42 [27]	0.42
Male sex	86%	84%	0.84
Blunt mechanism	83%	75%	0.70
ISS	36 [25]	27 [21]	0.12
Mortality	33%	19%	0.22
INR > 1.2	47%	21%	0.04
> 10 RBC	38%	28%	0.40

ISS, Injury Severity Score; INR, International Normalized Ratio; RBC, red blood cells

Continuous variables are reported as median with interquartile range [IQR]. Categorical variables are reported as percentages

In the ROTEM-period group, 35 of 43 patients (81%) had ROTEM testing performed. Four of the eight patients without ROTEM testing died in the emergency department from exsanguination after sustaining non-survivable injuries. Three of these eight patients sustained penetrating injuries that were associated with low ISS scores and stabilized without ROTEM-guided resuscitation.

**Fig. 1:**
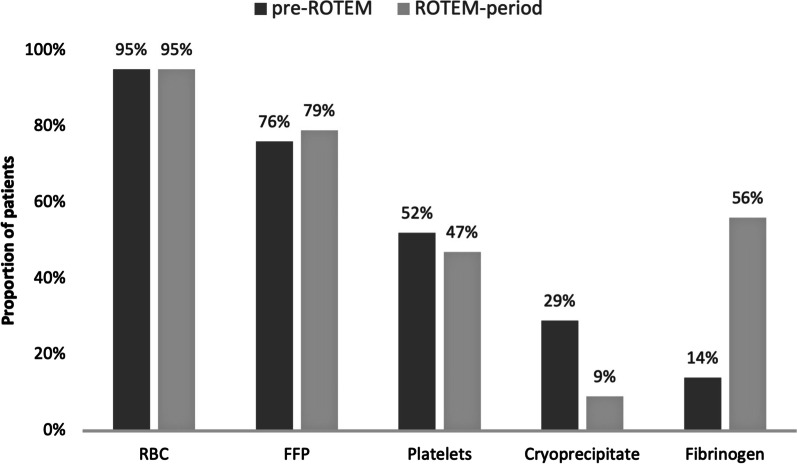
Percentage of patients transfused with 1 or more units per blood product

Comparing transfusion numbers, only 10 patients received RBC, FFP, platelet and cryoprecipitate: 6 patients in the pre-ROTEM group and four patients in the ROTEM-period. Thirty-one patients were transfused with RBC, FFP and platelets: 11 patients in the pre-ROTEM group and 20 in the ROTEM-period. Nineteen of these patients also received fibrinogen transfusion, all in the ROTEM-period. Before the introduction of ROTEM-guided transfusion, the median total blood product administration was 12 units [IQR 16], while in the ROTEM-period, the median total was 9 units [IQR 14]. The proportion of patients receiving one or more units of RBC was unchanged after the introduction of ROTEM (Fig. 1).

The use of fibrinogen concentrate was significantly greater in the ROTEM-period group compared to the pre-ROTEM group (*p* = 0.006). The percentage of patients receiving fibrinogen concentrate was four times greater (Table 2). The percentage of patients receiving any fibrinogen replacement (i.e. fibrinogen concentrate or cryoprecipitate) was also 20% higher; however, this was a non-significant increase (*p* = 0.13). A sensitivity analysis comparing pre-ROTEM patients and patients who were in the ROTEM-period group and had a ROTEM test done (Table 3) demonstrated that fibrinogen use remained greater (*p* < 0.001).

**Table 2:** Transfusion units within 24 h from admission for pre-ROTEM and ROTEM-period patients

Variable	Pre-ROTEM	ROTEM-period	*p* value
RBCs	6 [8]	7 [8]	0.85
FFP	3 [6]	3 [4]	0.86
Platelets	1 [3]	0 [2]	0.68
Cryoprecipitate	0 [1]	0 [0]	0.05
Fibrinogen	0 [0]	4 [4]	0.006

RBC, red blood cells; FFP, fresh frozen plasma

Continuous variables are reported as median with interquartile range [IQR]

**Table 3:** Transfusion units within 24 h from admission between pre-ROTEM, ROTEM-period (not done), and ROTEM-done patients

Variable	Pre-ROTEM	ROTEM-period (not done)	ROTEM-done	*p* value
RBCs	6 [8]	3.5 [5]	8 [8]	0.28
FFP	3 [6]	1 [4]	4 [4]	0.07
Platelets	1 [3]	0 [0]	1 [3]	0.32
Cryoprecipitate	0 [1]	0 [0]	0 [0]	0.10
Fibrinogen	0 [0]	0 [0]	4 [4]	< 0.001

Variables are reported as median with interquartile range [IQR]

After comparing ratios of FFP:RBC, platelets:RBC, fibrinogen concentrate:RBC, and any fibrinogen replacement:RBC (Table 4), only the ratios of fibrinogen concentrate:RBC (*p* < 0.001) and any fibrinogen replacement:RBC (*p* = 0.028) increased in the ROTEM-period group in patients receiving each blood product. Of the massive transfusion patients (10 or more units of packed RBCs in 24 h), there were eight patients in the pre-ROTEM group and 12 in the ROTEM-period group, of whom 11 received ROTEM-guided transfusion. Only the fibrinogen concentrate:RBC ratio was significantly greater in the ROTEM-period group (*p* = 0.03).

**Table 4:** Transfusion ratios within 24 h from admission in patients receiving each product

Variable	Pre-ROTEM	ROTEM-period	*p* value
FFP: RBCs	0.7 [0.35]: 1	0.55 [0.39]: 1	0.244
Platelets: RBCs	0.23 [0.08]: 1	0.19 [0.15]: 1	0.338
Fibrinogen (conc.): RBCs	0 [0]: 1	0.34 [0.51]: 1	< 0.001
Fibrinogen (any): RBCs	0 [0.13]: 1	0.32 [0.73]: 1	0.028

RBC, red blood cells; FFP, fresh frozen plasma; Fibrinogen (conc.), Fibrinogen Concentrate; Fibrinogen (any), Fibrinogen or Cryoprecipitate

Continuous variables are reported as median with interquartile range [IQR]

The overall mortality rate was 23.4%. Of these patients, all but two patients died from exsanguination within 24 h. The two patients who did not die from exsanguination died of non-survivable brain injuries. The cause of death was determined by patients’ most responsible physician. There was no significant difference in the in-hospital mortality rate between the pre-ROTEM and ROTEM-period groups (33% vs. 19%; *p* = 0.22). Similarly, there was no significant difference in mortality rates after excluding the two patients who sustained non-survivable brain injuries (26% vs. 19%; *p* = 0.50).

To further determine the effect of the implementation of ROTEM-guided transfusion, we compared the parameters found with conventional blood tests to those found with ROTEM. When compared with conventional coagulation tests, 74% (26/35) of patients showed fibrinogen deficiency with ROTEM parameters, while 37% (13/35) were found to have a low fibrinogen level in conventional blood tests. ROTEM parameters found 6% (2/35 patients) with thrombocytopenia, while conventional blood tests showed 26% (9/35 patients) with below normal platelet counts. 20% (7/35) of patients had a clotting factor deficiency on the ROTEM, which manifested as a prolonged EXTEM CT. The analogous blood test to identify a clotting factor deficiency is an INR greater than 1.2, which was found in 23% (8/35) of patients. An EXTEM ML30 greater than 10% was found in 6% (2/35) of patients, thus indicating hyperfibrinolysis.

## Discussion

The aim of this study was to evaluate the impact of ROTEM-guided transfusion on blood product usage and mortality of trauma patients at a Level 1 trauma center. The findings of this study demonstrate that ROTEM-guided transfusion primarily resulted in increased fibrinogen usage, with no significant difference in the transfusion of other blood components. ROTEM-guided transfusion was associated with an overall decreased mortality rate; however, this finding was non-significant (*p* = 0.22). The ROTEM test itself was more likely to identify fibrinogen deficiency than standard blood tests. There was no clinically relevant difference when examining ROTEM and standard blood test results for thrombocytopenia. The overall compliance for ROTEM testing in massive hemorrhage protocols for trauma patients at this institution was 81%. Non-compliance was primarily seen in non-survivable injuries where ROTEM-guided transfusion was not deemed appropriate by the clinical team.

Like other studies examining the usefulness and applications of ROTEM within hospital settings, this study provides evidence to support targeted blood product usage guided by ROTEM in trauma patients [17, 20, 21]. Furthermore, this study is unique in its description and analysis of individual blood components. The primary finding of this study that is scarcely mentioned in other literature on ROTEM-guided transfusion in trauma patients is that ROTEM-guided transfusion resulted in increased fibrinogen concentrate usage and higher ratios of fibrinogen replacement in relation to blood components, compared to transfusion guided by standard laboratory tests. However, the increased fibrinogen usage may not have been necessary in this context, considering that the mortality rates between groups were similar. Nonetheless, multiple studies have shown that patients with acquired fibrinogen deficits are more likely to suffer from morbidity or mortality in settings of trauma, cardiac surgery, and postpartum hemorrhage [22–25]. Trauma-induced coagulopathy has also been characterized by acquired hypofibrinogenemia as the earliest finding of coagulopathy. Thus, testing to promptly identify fibrinogen deficiencies early in the resuscitation process is key to treating coagulopathy. Differences in blood product usage with ROTEM were not shown for RBCs, FFP, platelets, or cryoprecipitate. At our center, increased cryoprecipitate usage was not observed as use of fibrinogen concentrate provided a more consistent replacement strategy for fibrinogen deficiencies identified on ROTEM testing. Likewise, this study found no significant difference in mortality rates in ROTEM-period trauma patients, which is a finding both supported and opposed by the current literature. Our single center study was likely underpowered to find differences in mortality.

Given that ROTEM-guided transfusion was performed on 81% of eligible patients, a sensitivity analysis was conducted examining pre-ROTEM patients to patients with ROTEM testing performed after implementation (i.e. excluding ROTEM-period patients who did not have ROTEM-guided transfusion). The results were in agreement with the analysis done on the original cohort: only fibrinogen concentrate use was significantly higher compared to pre-ROTEM patients (Table 3). This finding suggested that the non-compliance rate seen in this institution did not extensively affect the study results. Therefore there was no need for further sensitivity analyses or stratification.

The presented findings of this study, and hence their relevance to the current literature, may have been impacted due to our limited sample size in both the pre-ROTEM and ROTEM-period cohorts. In addition, the findings of our study may have been affected by confounders given its retrospective observational design. The association of ROTEM with utilization differences cannot be construed as causative, as whether transfusion occurred as a result of ROTEM or occurred after ROTEM was not considered in this study. The inclusion of two temporally distinct cohorts comprised of patients with unique injuries and traumatic presentations, potentially producing differences in practice from provider preferences or advances in health-services provision, may have impacted our findings. We attempted to minimize selection bias through inclusion of consecutive eligible trauma patients with comparable ages, sex, injury mechanisms, and ISS (Table 1).

ROTEM-guided transfusion is a novel approach that can assist clinicians with managing massive hemorrhage of trauma patients. We recommend that further examination of ROTEM-guided transfusion be conducted to ascertain its benefits as a point-of-care tool for trauma patients requiring blood product administration. Future studies would benefit from a larger sample size and randomization or matching procedures to control for confounders. In addition, the investigation of cost–benefit analyses and multi-site studies should be conducted to examine feasibility and effectiveness of this novel mode of transfusion for trauma patients.

## Supplementary Information

**Additional file 1**. **Fig. S1** Institutional ROTEM-guided bleeding management pathway for trauma.

## Data Availability

Due to institutional policies regulating the sharing of potentially identifiable health data, interested individuals should contact the corresponding author (mina.salehi@vch.ca) to be connected with the data steward of the health institution.

## Abbreviations

A10: Amplitude at 10 min
APTT: Activated partial thromboplastin time
CT: Clotting time
FFP: Fresh frozen plasma
INR: International normalized ratio
IQR: Interquartile range
ISS: Injury severity score
MCF: Maximum clot firmness
ML: Maximum lysis
PT: Prothrombin time
RBC: Red blood cell
ROTEM: Rotational thromboelastometry
SD: Standard deviation
TEP: Trauma exsanguination protocol
