# Supplementary figures and images for: Guided blood transfusion of trauma patients with rotational thromboelastometry: a single-center cohort study
Source: World J Emerg Surg. 2023 Jul 1;18:40. doi: 10.1186/s13017-023-00508-5 (PMC10315024; doi:10.1186/s13017-023-00508-5)

**
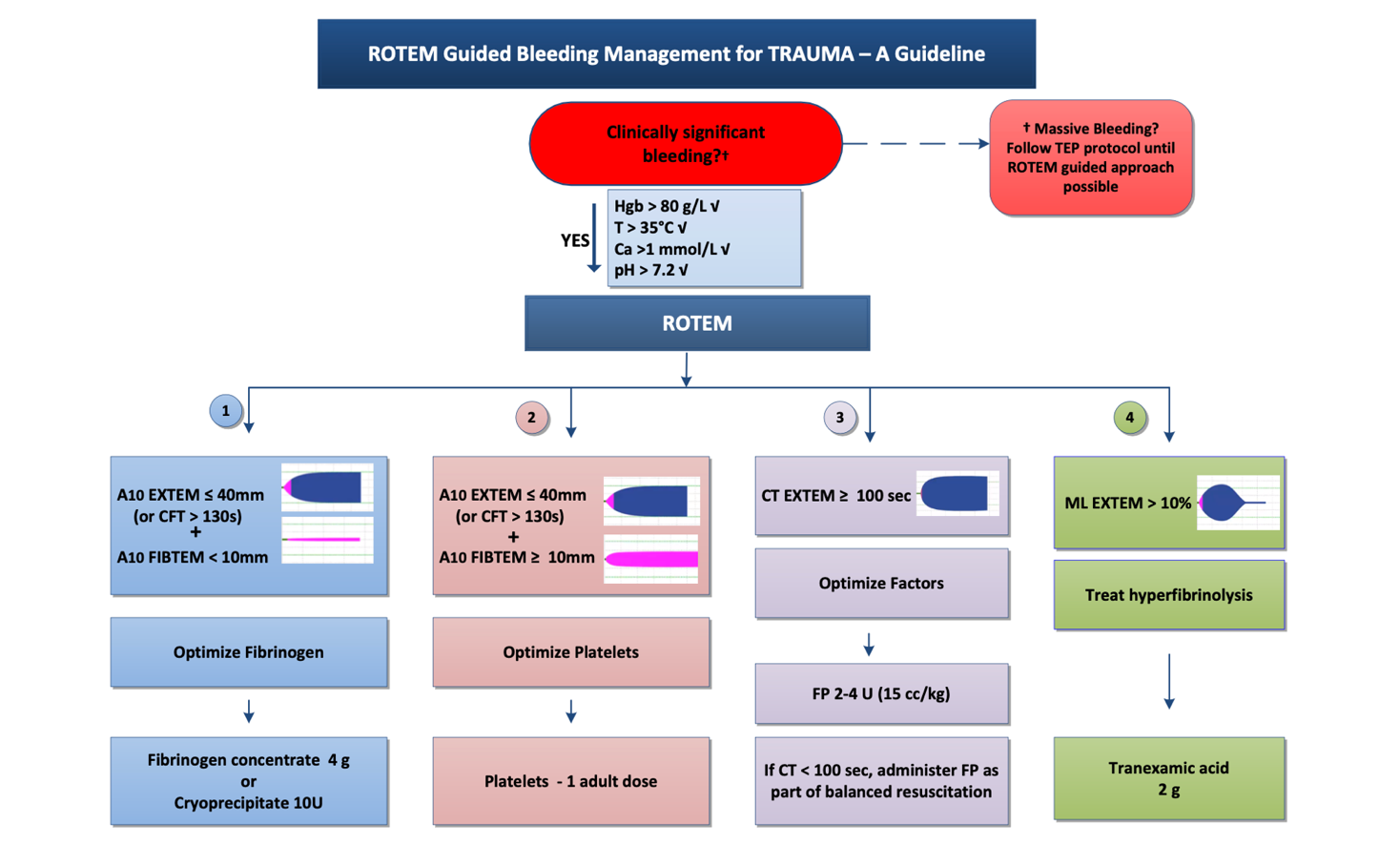
**

**Supplemental Figure 1.** Institutional ROTEM-guided bleeding management pathway for trauma.

Supplement: Supplementary file 1 — Additional file 1. Fig. S1 Institutional ROTEM-guided bleeding management pathway for trauma. [file 13017_2023_508_MOESM1_ESM.docx]
